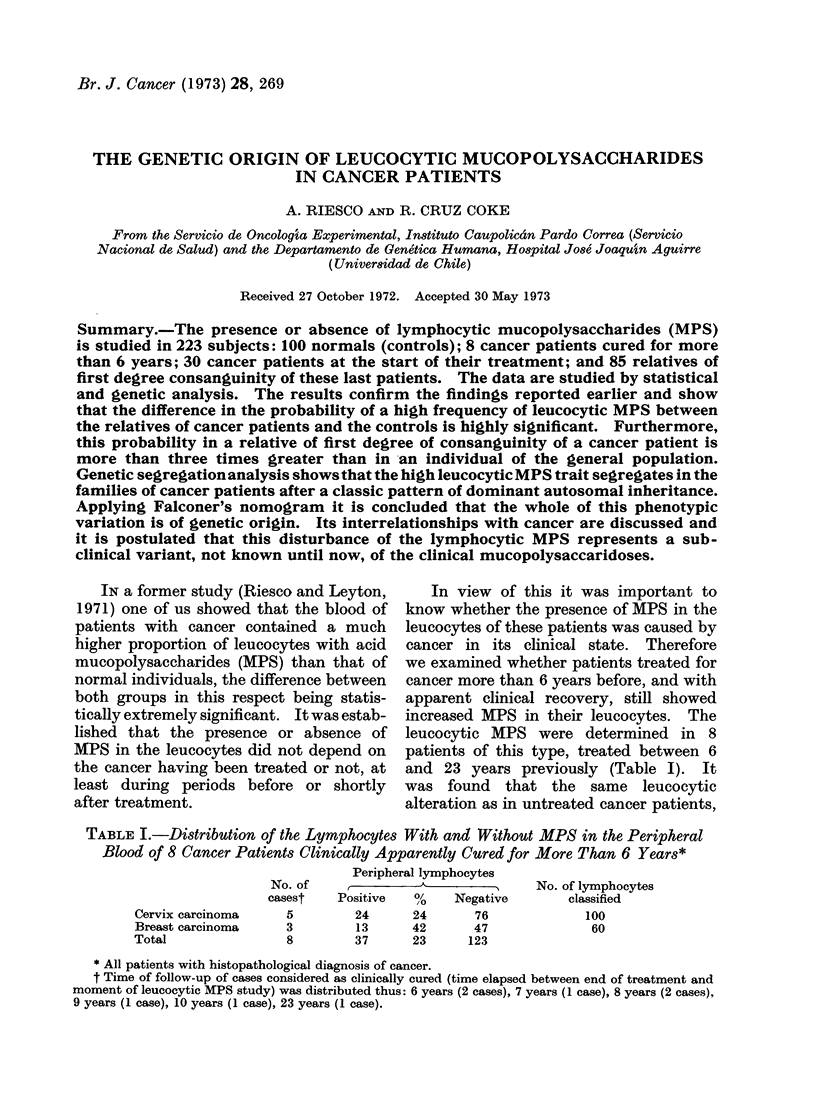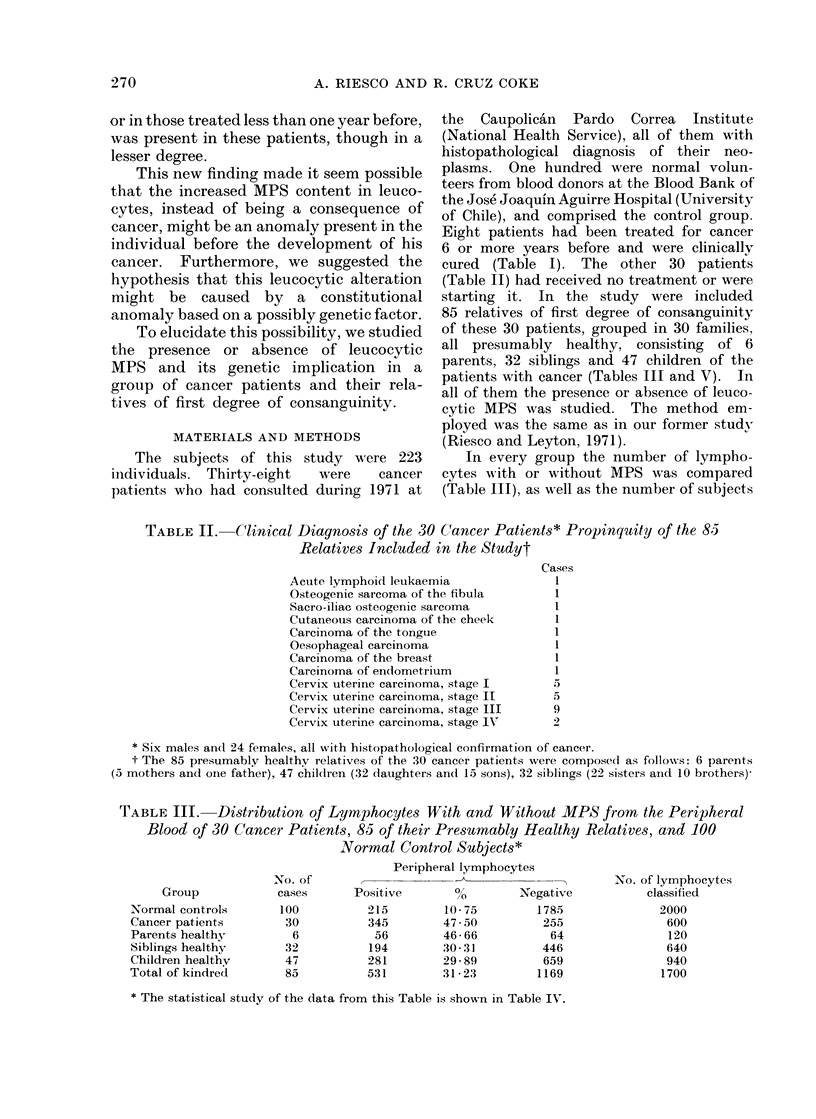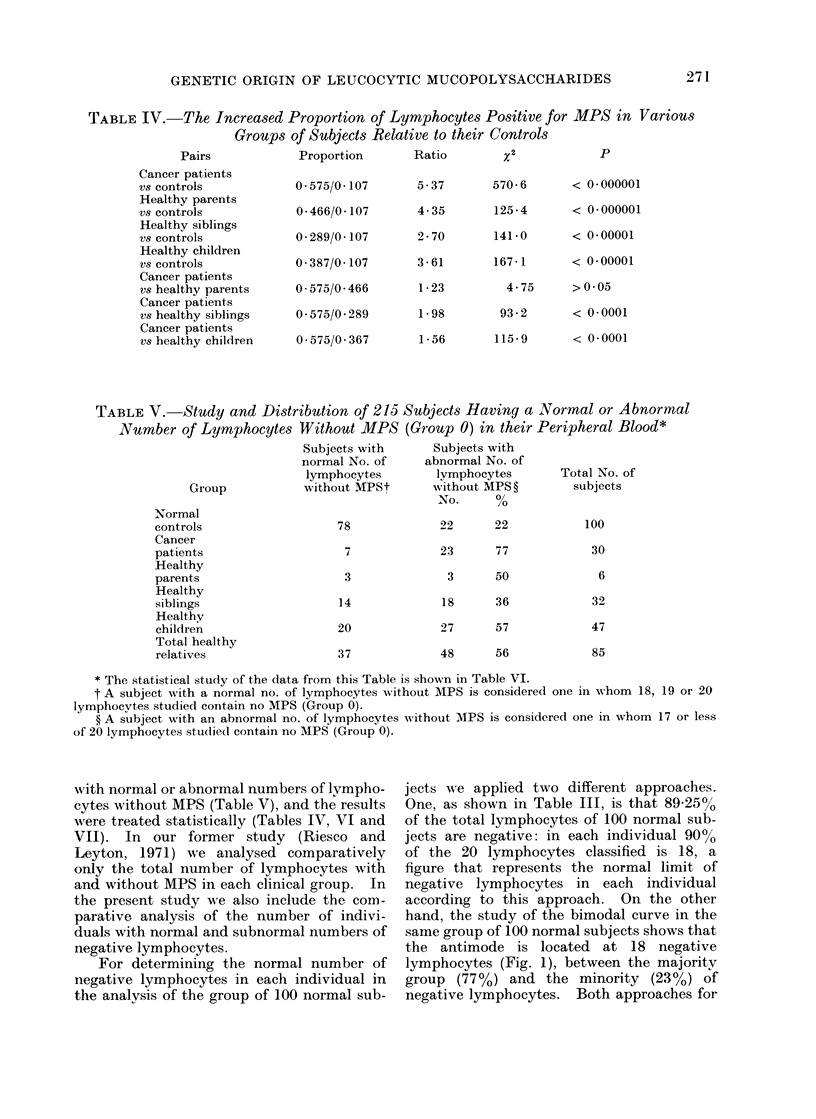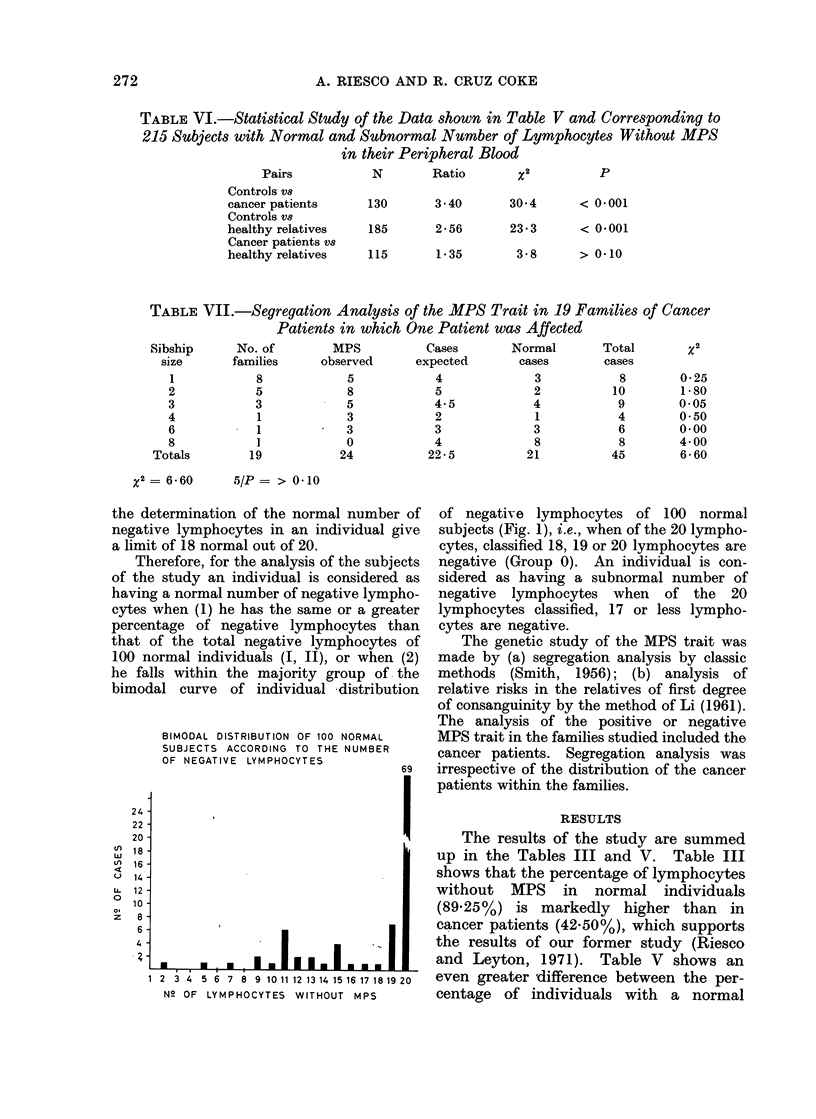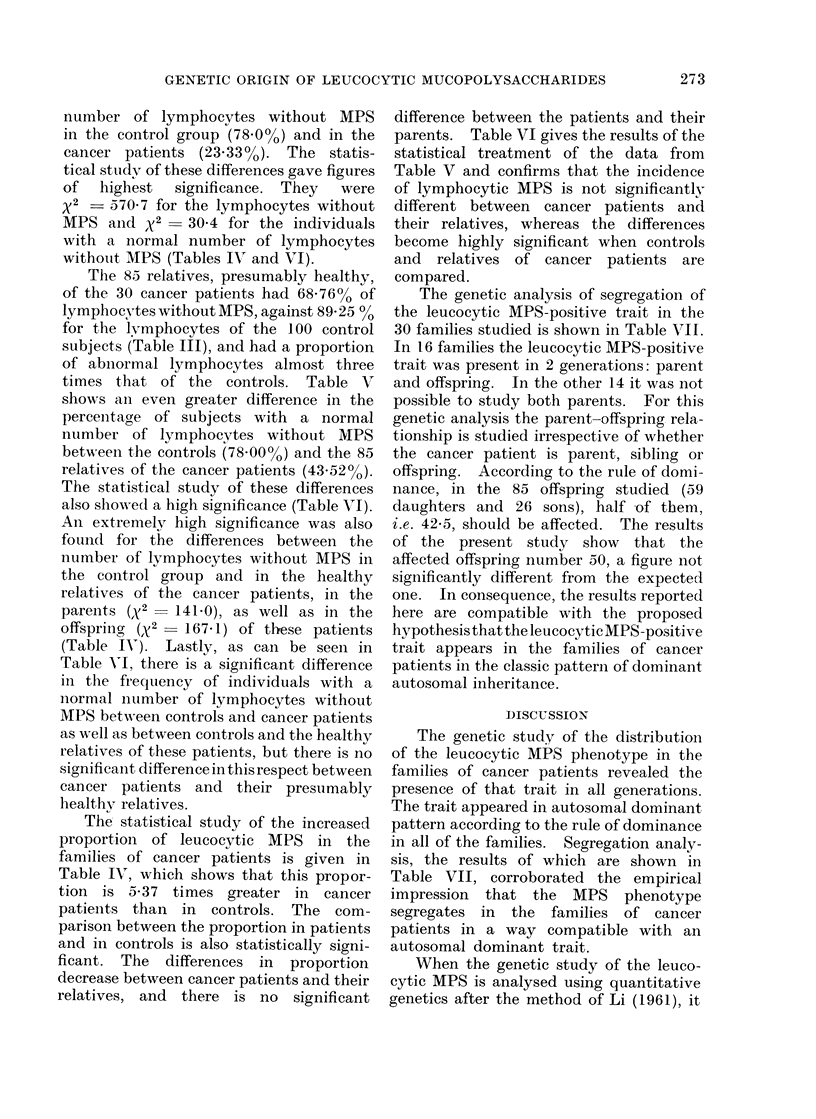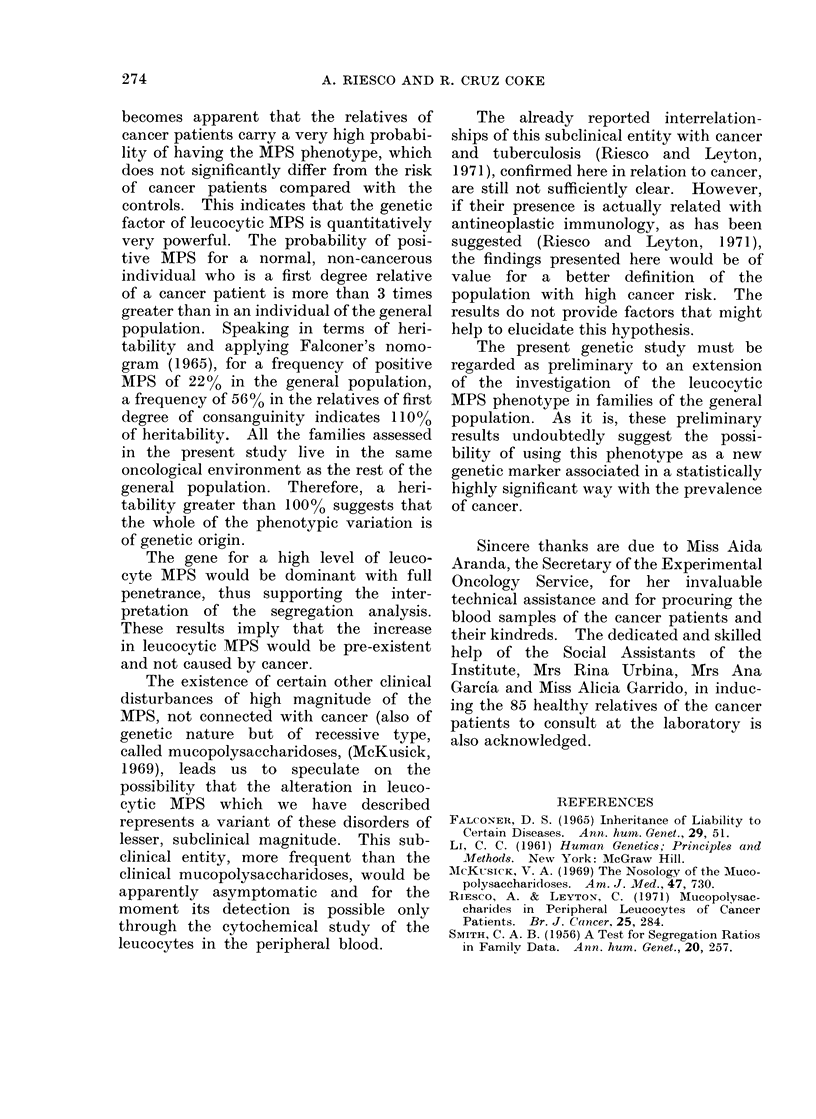# The Genetic Origin of Leucocytic Mucopolysaccharides in Cancer Patients

**DOI:** 10.1038/bjc.1973.147

**Published:** 1973-09

**Authors:** A. Riesco, R. Cruz Coke

## Abstract

The presence or absence of lymphocytic mucopolysaccharides (MPS) is studied in 223 subjects: 100 normals (controls); 8 cancer patients cured for more than 6 years; 30 cancer patients at the start of their treatment; and 85 relatives of first degree consanguinity of these last patients. The data are studied by statistical and genetic analysis. The results confirm the findings reported earlier and show that the difference in the probability of a high frequency of leucocytic MPS between the relatives of cancer patients and the controls is highly significant. Furthermore, this probability in a relative of first degree of consanguinity of a cancer patient is more than three times greater than in an individual of the general population. Genetic segregation analysis shows that the high leucocytic MPS trait segregates in the families of cancer patients after a classic pattern of dominant autosomal inheritance. Applying Falconer's nomogram it is concluded that the whole of this phenotypic variation is of genetic origin. Its interrelationships with cancer are discussed and it is postulated that this disturbance of the lymphocytic MPS represents a subclinical variant, not known until now, of the clinical mucopolysaccaridoses.


Br. J. Cancer (1973) 28, 269

### THE GENETIC ORIGIN OF LEUCOCYTIC MUCOPOLYSACCHARIDES IN CANCER PATIENTS

A. RIESCO AND R. CRUZ COKE

*From the Servicio de Oncología Experimental, Instituto Caupolicán Pardo Correa (Servicio Nacional de Salud) and the Departamento de Genética Humana, Hospital José Joaquín Aguirre (Universidad de Chile)*

Received 27 October 1972. Accepted 30 May 1973

**Summary.—The presence or absence of lymphocytic mucopolysaccharides (MPS) is studied in 223 subjects: 100 normals (controls); 8 cancer patients cured for more than 6 years; 30 cancer patients at the start of their treatment; and 85 relatives of first degree consanguinity of these last patients. The data are studied by statistical and genetic analysis. The results confirm the findings reported earlier and show that the difference in the probability of a high frequency of leucocytic MPS between the relatives of cancer patients and the controls is highly significant. Furthermore, this probability in a relative of first degree of consanguinity of a cancer patient is more than three times greater than in an individual of the general population. Genetic segregation analysis shows that the high leucocytic MPS trait segregates in the families of cancer patients after a classic pattern of dominant autosomal inheritance. Applying Falconer's nomogram it is concluded that the whole of this phenotypic variation is of genetic origin. Its interrelationships with cancer are discussed and it is postulated that this disturbance of the lymphocytic MPS represents a sub-clinical variant, not known until now, of the clinical mucopolysaccaridoses.**

In a former study (Riesco and Leyton, 1971) one of us showed that the blood of patients with cancer contained a much higher proportion of leucocytes with acid mucopolysaccharides (MPS) than that of normal individuals, the difference between both groups in this respect being statistically extremely significant. It was established that the presence or absence of MPS in the leucocytes did not depend on the cancer having been treated or not, at least during periods before or shortly after treatment.

In view of this it was important to know whether the presence of MPS in the leucocytes of these patients was caused by cancer in its clinical state. Therefore we examined whether patients treated for cancer more than 6 years before, and with apparent clinical recovery, still showed increased MPS in their leucocytes. The leucocytic MPS were determined in 8 patients of this type, treated between 6 and 23 years previously (Table I). It was found that the same leucocytic alteration as in untreated cancer patients,

TABLE I.—*Distribution of the Lymphocytes With and Without MPS in the Peripheral Blood of 8 Cancer Patients Clinically Apparently Cured for More Than 6 Years**

| | No. of cases† | Peripheral lymphocytes | | | No. of lymphocytes classified |
|---|---|---|---|---|---|
| | | Positive | % | Negative | |
| Cervix carcinoma | 5 | 24 | 24 | 76 | 100 |
| Breast carcinoma | 3 | 13 | 42 | 47 | 60 |
| Total | 8 | 37 | 23 | 123 | |

* All patients with histopathological diagnosis of cancer.

† Time of follow-up of cases considered as clinically cured (time elapsed between end of treatment and moment of leucocytic MPS study) was distributed thus: 6 years (2 cases), 7 years (1 case), 8 years (2 cases), 9 years (1 case), 10 years (1 case), 23 years (1 case).

or in those treated less than one year before, was present in these patients, though in a lesser degree.

This new finding made it seem possible that the increased MPS content in leucocytes, instead of being a consequence of cancer, might be an anomaly present in the individual before the development of his cancer. Furthermore, we suggested the hypothesis that this leucocytic alteration might be caused by a constitutional anomaly based on a possibly genetic factor.

To elucidate this possibility, we studied the presence or absence of leucocytic MPS and its genetic implication in a group of cancer patients and their relatives of first degree of consanguinity.

### MATERIALS AND METHODS

The subjects of this study were 223 individuals. Thirty-eight were cancer patients who had consulted during 1971 at the Caupolicán Pardo Correa Institute (National Health Service), all of them with histopathological diagnosis of their neoplasms. One hundred were normal volunteers from blood donors at the Blood Bank of the José Joaquín Aguirre Hospital (University of Chile), and comprised the control group. Eight patients had been treated for cancer 6 or more years before and were clinically cured (Table I). The other 30 patients (Table II) had received no treatment or were starting it. In the study were included 85 relatives of first degree of consanguinity of these 30 patients, grouped in 30 families, all presumably healthy, consisting of 6 parents, 32 siblings and 47 children of the patients with cancer (Tables III and V). In all of them the presence or absence of leucocytic MPS was studied. The method employed was the same as in our former study (Riesco and Leyton, 1971).

In every group the number of lymphocytes with or without MPS was compared (Table III), as well as the number of subjects

TABLE II.—*Clinical Diagnosis of the 30 Cancer Patients* Propinquity of the 85 Relatives Included in the Study†*

| | Cases |
|---|---|
| Acute lymphoid leukaemia | 1 |
| Osteogenic sarcoma of the fibula | 1 |
| Sacro-iliac osteogenic sarcoma | 1 |
| Cutaneous carcinoma of the cheek | 1 |
| Carcinoma of the tongue | 1 |
| Oesophageal carcinoma | 1 |
| Carcinoma of the breast | 1 |
| Carcinoma of endometrium | 1 |
| Cervix uterine carcinoma, stage I | 5 |
| Cervix uterine carcinoma, stage II | 5 |
| Cervix uterine carcinoma, stage III | 9 |
| Cervix uterine carcinoma, stage IV | 2 |

* Six males and 24 females, all with histopathological confirmation of cancer.

† The 85 presumably healthy relatives of the 30 cancer patients were composed as follows: 6 parents (5 mothers and one father), 47 children (32 daughters and 15 sons), 32 siblings (22 sisters and 10 brothers).

TABLE III.—*Distribution of Lymphocytes With and Without MPS from the Peripheral Blood of 30 Cancer Patients, 85 of their Presumably Healthy Relatives, and 100 Normal Control Subjects**

| Group | No. of cases | Peripheral lymphocytes | | | No. of lymphocytes classified |
|---|---|---|---|---|---|
| | | Positive | % | Negative | |
| Normal controls | 100 | 215 | 10·75 | 1785 | 2000 |
| Cancer patients | 30 | 345 | 47·50 | 255 | 600 |
| Parents healthy | 6 | 56 | 46·66 | 64 | 120 |
| Siblings healthy | 32 | 194 | 30·31 | 446 | 640 |
| Children healthy | 47 | 281 | 29·89 | 659 | 940 |
| Total of kindred | 85 | 531 | 31·23 | 1169 | 1700 |

* The statistical study of the data from this Table is shown in Table IV.

TABLE IV.—*The Increased Proportion of Lymphocytes Positive for MPS in Various Groups of Subjects Relative to their Controls*

| Pairs | Proportion | Ratio | $\chi^2$ | *P* |
|---|---|---|---|---|
| Cancer patients *vs* controls | 0·575/0·107 | 5·37 | 570·6 | < 0·000001 |
| Healthy parents *vs* controls | 0·466/0·107 | 4·35 | 125·4 | < 0·000001 |
| Healthy siblings *vs* controls | 0·289/0·107 | 2·70 | 141·0 | < 0·00001 |
| Healthy children *vs* controls | 0·387/0·107 | 3·61 | 167·1 | < 0·00001 |
| Cancer patients *vs* healthy parents | 0·575/0·466 | 1·23 | 4·75 | > 0·05 |
| Cancer patients *vs* healthy siblings | 0·575/0·289 | 1·98 | 93·2 | < 0·0001 |
| Cancer patients *vs* healthy children | 0·575/0·367 | 1·56 | 115·9 | < 0·0001 |

TABLE V.—*Study and Distribution of 215 Subjects Having a Normal or Abnormal Number of Lymphocytes Without MPS (Group 0) in their Peripheral Blood**

| Group | Subjects with normal No. of lymphocytes without MPS† | Subjects with abnormal No. of lymphocytes without MPS§ | | Total No. of subjects |
|---|---|---|---|---|
| | | No. | % | |
| Normal controls | 78 | 22 | 22 | 100 |
| Cancer patients | 7 | 23 | 77 | 30 |
| Healthy parents | 3 | 3 | 50 | 6 |
| Healthy siblings | 14 | 18 | 36 | 32 |
| Healthy children | 20 | 27 | 57 | 47 |
| Total healthy relatives | 37 | 48 | 56 | 85 |

* The statistical study of the data from this Table is shown in Table VI.

† A subject with a normal no. of lymphocytes without MPS is considered one in whom 18, 19 or 20 lymphocytes studied contain no MPS (Group 0).

§ A subject with an abnormal no. of lymphocytes without MPS is considered one in whom 17 or less of 20 lymphocytes studied contain no MPS (Group 0).

with normal or abnormal numbers of lymphocytes without MPS (Table V), and the results were treated statistically (Tables IV, VI and VII). In our former study (Riesco and Leyton, 1971) we analysed comparatively only the total number of lymphocytes with and without MPS in each clinical group. In the present study we also include the comparative analysis of the number of individuals with normal and subnormal numbers of negative lymphocytes.

For determining the normal number of negative lymphocytes in each individual in the analysis of the group of 100 normal subjects we applied two different approaches. One, as shown in Table III, is that 89·25% of the total lymphocytes of 100 normal subjects are negative: in each individual 90% of the 20 lymphocytes classified is 18, a figure that represents the normal limit of negative lymphocytes in each individual according to this approach. On the other hand, the study of the bimodal curve in the same group of 100 normal subjects shows that the antimode is located at 18 negative lymphocytes (Fig. 1), between the majority group (77%) and the minority (23%) of negative lymphocytes. Both approaches for

TABLE VI.—*Statistical Study of the Data shown in Table V and Corresponding to 215 Subjects with Normal and Subnormal Number of Lymphocytes Without MPS in their Peripheral Blood*

| Pairs | N | Ratio | $\chi^2$ | $P$ |
|---|---|---|---|---|
| Controls *vs* cancer patients | 130 | 3·40 | 30·4 | < 0·001 |
| Controls *vs* healthy relatives | 185 | 2·56 | 23·3 | < 0·001 |
| Cancer patients *vs* healthy relatives | 115 | 1·35 | 3·8 | > 0·10 |

TABLE VII.—*Segregation Analysis of the MPS Trait in 19 Families of Cancer Patients in which One Patient was Affected*

| Sibship size | No. of families | MPS observed | Cases expected | Normal cases | Total cases | $\chi^2$ |
|---|---|---|---|---|---|---|
| 1 | 8 | 5 | 4 | 3 | 8 | 0·25 |
| 2 | 5 | 8 | 5 | 2 | 10 | 1·80 |
| 3 | 3 | 5 | 4·5 | 4 | 9 | 0·05 |
| 4 | 1 | 3 | 2 | 1 | 4 | 0·50 |
| 6 | 1 | 3 | 3 | 3 | 6 | 0·00 |
| 8 | 1 | 0 | 4 | 8 | 8 | 4·00 |
| Totals | 19 | 24 | 22·5 | 21 | 45 | 6·60 |

$\chi^2 = 6\cdot60$ $5/P = > 0\cdot10$

the determination of the normal number of negative lymphocytes in an individual give a limit of 18 normal out of 20.

Therefore, for the analysis of the subjects of the study an individual is considered as having a normal number of negative lymphocytes when (1) he has the same or a greater percentage of negative lymphocytes than that of the total negative lymphocytes of 100 normal individuals (I, II), or when (2) he falls within the majority group of the bimodal curve of individual distribution of negative lymphocytes of 100 normal subjects (Fig. 1), *i.e.*, when of the 20 lymphocytes, classified 18, 19 or 20 lymphocytes are negative (Group 0). An individual is considered as having a subnormal number of negative lymphocytes when of the 20 lymphocytes classified, 17 or less lymphocytes are negative.

The genetic study of the MPS trait was made by (a) segregation analysis by classic methods (Smith, 1956); (b) analysis of relative risks in the relatives of first degree of consanguinity by the method of Li (1961). The analysis of the positive or negative MPS trait in the families studied included the cancer patients. Segregation analysis was irrespective of the distribution of the cancer patients within the families.

### RESULTS

The results of the study are summed up in the Tables III and V. Table III shows that the percentage of lymphocytes without MPS in normal individuals (89·25%) is markedly higher than in cancer patients (42·50%), which supports the results of our former study (Riesco and Leyton, 1971). Table V shows an even greater difference between the percentage of individuals with a normal number of lymphocytes without MPS in the control group (78·0%) and in the cancer patients (23·33%). The statistical study of these differences gave figures of highest significance. They were $\chi^2 = 570 \cdot 7$ for the lymphocytes without MPS and $\chi^2 = 30 \cdot 4$ for the individuals with a normal number of lymphocytes without MPS (Tables IV and VI).

The 85 relatives, presumably healthy, of the 30 cancer patients had 68·76% of lymphocytes without MPS, against 89·25 % for the lymphocytes of the 100 control subjects (Table III), and had a proportion of abnormal lymphocytes almost three times that of the controls. Table V shows an even greater difference in the percentage of subjects with a normal number of lymphocytes without MPS between the controls (78·00%) and the 85 relatives of the cancer patients (43·52%). The statistical study of these differences also showed a high significance (Table VI). An extremely high significance was also found for the differences between the number of lymphocytes without MPS in the control group and in the healthy relatives of the cancer patients, in the parents ($\chi^2 = 141 \cdot 0$), as well as in the offspring ($\chi^2 = 167 \cdot 1$) of these patients (Table IV). Lastly, as can be seen in Table VI, there is a significant difference in the frequency of individuals with a normal number of lymphocytes without MPS between controls and cancer patients as well as between controls and the healthy relatives of these patients, but there is no significant difference in this respect between cancer patients and their presumably healthy relatives.

The statistical study of the increased proportion of leucocytic MPS in the families of cancer patients is given in Table IV, which shows that this proportion is 5·37 times greater in cancer patients than in controls. The comparison between the proportion in patients and in controls is also statistically significant. The differences in proportion decrease between cancer patients and their relatives, and there is no significant difference between the patients and their parents. Table VI gives the results of the statistical treatment of the data from Table V and confirms that the incidence of lymphocytic MPS is not significantly different between cancer patients and their relatives, whereas the differences become highly significant when controls and relatives of cancer patients are compared.

The genetic analysis of segregation of the leucocytic MPS-positive trait in the 30 families studied is shown in Table VII. In 16 families the leucocytic MPS-positive trait was present in 2 generations: parent and offspring. In the other 14 it was not possible to study both parents. For this genetic analysis the parent–offspring relationship is studied irrespective of whether the cancer patient is parent, sibling or offspring. According to the rule of dominance, in the 85 offspring studied (59 daughters and 26 sons), half of them, *i.e.* 42·5, should be affected. The results of the present study show that the affected offspring number 50, a figure not significantly different from the expected one. In consequence, the results reported here are compatible with the proposed hypothesis that the leucocytic MPS-positive trait appears in the families of cancer patients in the classic pattern of dominant autosomal inheritance.

### DISCUSSION

The genetic study of the distribution of the leucocytic MPS phenotype in the families of cancer patients revealed the presence of that trait in all generations. The trait appeared in autosomal dominant pattern according to the rule of dominance in all of the families. Segregation analysis, the results of which are shown in Table VII, corroborated the empirical impression that the MPS phenotype segregates in the families of cancer patients in a way compatible with an autosomal dominant trait.

When the genetic study of the leucocytic MPS is analysed using quantitative genetics after the method of Li (1961), it becomes apparent that the relatives of cancer patients carry a very high probability of having the MPS phenotype, which does not significantly differ from the risk of cancer patients compared with the controls. This indicates that the genetic factor of leucocytic MPS is quantitatively very powerful. The probability of positive MPS for a normal, non-cancerous individual who is a first degree relative of a cancer patient is more than 3 times greater than in an individual of the general population. Speaking in terms of heritability and applying Falconer's nomogram (1965), for a frequency of positive MPS of 22% in the general population, a frequency of 56% in the relatives of first degree of consanguinity indicates 110% of heritability. All the families assessed in the present study live in the same oncological environment as the rest of the general population. Therefore, a heritability greater than 100% suggests that the whole of the phenotypic variation is of genetic origin.

The gene for a high level of leucocyte MPS would be dominant with full penetrance, thus supporting the interpretation of the segregation analysis. These results imply that the increase in leucocytic MPS would be pre-existent and not caused by cancer.

The existence of certain other clinical disturbances of high magnitude of the MPS, not connected with cancer (also of genetic nature but of recessive type, called mucopolysaccharidoses, (McKusick, 1969), leads us to speculate on the possibility that the alteration in leucocytic MPS which we have described represents a variant of these disorders of lesser, subclinical magnitude. This subclinical entity, more frequent than the clinical mucopolysaccharidoses, would be apparently asymptomatic and for the moment its detection is possible only through the cytochemical study of the leucocytes in the peripheral blood.

The already reported interrelationships of this subclinical entity with cancer and tuberculosis (Riesco and Leyton, 1971), confirmed here in relation to cancer, are still not sufficiently clear. However, if their presence is actually related with antineoplastic immunology, as has been suggested (Riesco and Leyton, 1971), the findings presented here would be of value for a better definition of the population with high cancer risk. The results do not provide factors that might help to elucidate this hypothesis.

The present genetic study must be regarded as preliminary to an extension of the investigation of the leucocytic MPS phenotype in families of the general population. As it is, these preliminary results undoubtedly suggest the possibility of using this phenotype as a new genetic marker associated in a statistically highly significant way with the prevalence of cancer.

Sincere thanks are due to Miss Aida Aranda, the Secretary of the Experimental Oncology Service, for her invaluable technical assistance and for procuring the blood samples of the cancer patients and their kindreds. The dedicated and skilled help of the Social Assistants of the Institute, Mrs Rina Urbina, Mrs Ana García and Miss Alicia Garrido, in inducing the 85 healthy relatives of the cancer patients to consult at the laboratory is also acknowledged.

### REFERENCES

Falconer, D. S. (1965) Inheritance of Liability to Certain Diseases. *Ann. hum. Genet.*, **29**, 51.

Li, C. C. (1961) *Human Genetics; Principles and Methods.* New York: McGraw Hill.

McKusick, V. A. (1969) The Nosology of the Mucopolysaccharidoses. *Am. J. Med.*, **47**, 730.

Riesco, A. & Leyton, C. (1971) Mucopolysaccharides in Peripheral Leucocytes of Cancer Patients. *Br. J. Cancer*, **25**, 284.

Smith, C. A. B. (1956) A Test for Segregation Ratios in Family Data. *Ann. hum. Genet.*, **20**, 257.